# 3-(4,6-Dichloro-1,3,5-triazin-2-yl)-2,2-dimethyl-1,3-oxazolidine

**DOI:** 10.1107/S1600536812026578

**Published:** 2012-06-20

**Authors:** Ye-cheng Zou, Zhi-yong Hu, Duan-lin Cao

**Affiliations:** a School of Chemical Engineering and Environment, North University of China, Taiyuan, People’s Republic of China

## Abstract

In the title compound, C_8_H_10_Cl_2_N_4_O, the dichloro-substituted triazine ring and the quasi-plane of the five-membered dimethyl-substituted oxazolidine unit, in which the O atom lies 0.228 (1) Å out of the least-squares plane, are close to being coplanar [dihedral angle = 4.99 (10)°]. In the crystal, mol­ecules are linked by inter­molecular C—H⋯Cl inter­actions, forming chains extend along the *a* axis. Also present are weak π–π inter­actions between triazine rings [minimum ring centroid separation = 3.7427 (11) Å].

## Related literature

For the properties of 1,3,5-triazines, see: Xue *et al.* (2011 ▶); Zhao *et al.* (2010 ▶). For the chemistry and synthesis of the title compound, see: Li *et al.* (2010 ▶); Yang *et al.* (2010 ▶); Rankin *et al.* (2002 ▶).
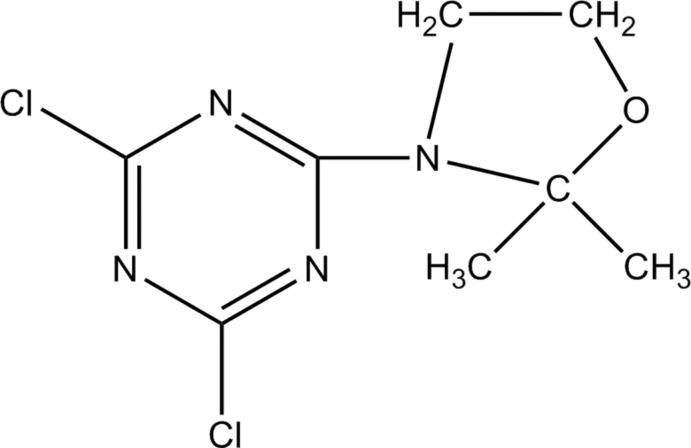


## Experimental

### 

#### Crystal data

C_8_H_10_Cl_2_N_4_O
*M* *_r_* = 249.10
Monoclinic, 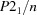

*a* = 8.1943 (10) Å
*b* = 11.0948 (17) Å
*c* = 11.8333 (18) Å
β = 94.383 (14)°
*V* = 1072.7 (3) Å^3^
*Z* = 4
Mo *K*α radiation
μ = 0.58 mm^−1^
*T* = 113 K
0.20 × 0.20 × 0.06 mm

#### Data collection

Rigaku Saturn724 CCD-detector diffractometer
Absorption correction: multi-scan (*SADABS*; Sheldrick, 1996 ▶) *T*_min_ = 0.892, *T*_max_ = 0.966
13220 measured reflections
2547 independent reflections
1592 reflections with *I* > 2σ(*I*)
*R*_int_ = 0.058

#### Refinement

*R*[*F* ^2^ > 2σ(*F* ^2^)] = 0.038
*wR*(*F* ^2^) = 0.088
*S* = 0.97
2547 reflections
138 parameters
H-atom parameters constrained
Δρ_max_ = 0.40 e Å^−3^
Δρ_min_ = −0.40 e Å^−3^

### 

Data collection: *CrystalClear* (Rigaku/MSC, 2000 ▶); cell refinement: *CrystalClear*; data reduction: *CrystalClear*; program(s) used to solve structure: *SHELXS97* (Sheldrick, 2008 ▶); program(s) used to refine structure: *SHELXL97* (Sheldrick, 2008 ▶); molecular graphics: *SHELXTL* (Sheldrick, 2008 ▶); software used to prepare material for publication: *CrystalStructure* (Rigaku/MSC, 2000 ▶).

## Supplementary Material

Crystal structure: contains datablock(s) I, global. DOI: 10.1107/S1600536812026578/zs2206sup1.cif

Structure factors: contains datablock(s) I. DOI: 10.1107/S1600536812026578/zs2206Isup2.hkl

Supplementary material file. DOI: 10.1107/S1600536812026578/zs2206Isup3.cml

Additional supplementary materials:  crystallographic information; 3D view; checkCIF report

## Figures and Tables

**Table 1:** Hydrogen-bond geometry (Å, °)

*D*—H⋯*A*	*D*—H	H⋯*A*	*D*⋯*A*	*D*—H⋯*A*
C2—H2*A*⋯Cl2^i^	0.99	2.78	3.522 (2)	132

Symmetry code: (i) 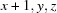.

**Fig. 1.**
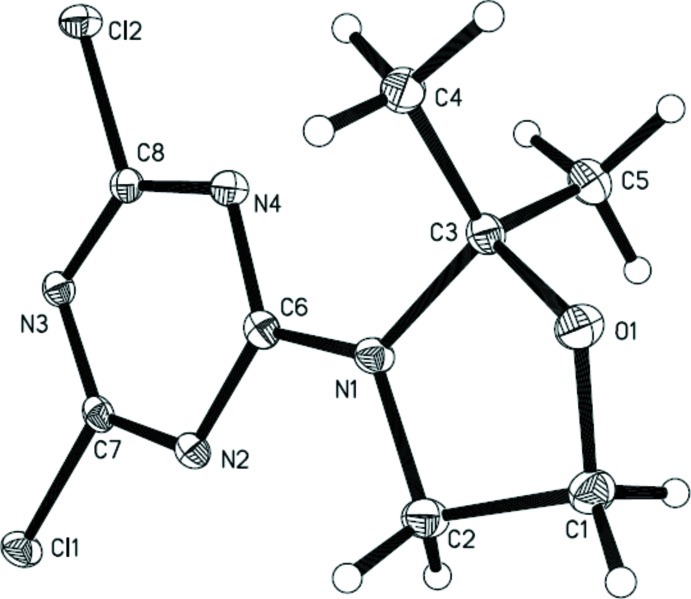
The molecular structure and atom-numbering scheme for the title compound, with atoms shown as 50% probability displacement ellipsoids.

**Fig. 2.**
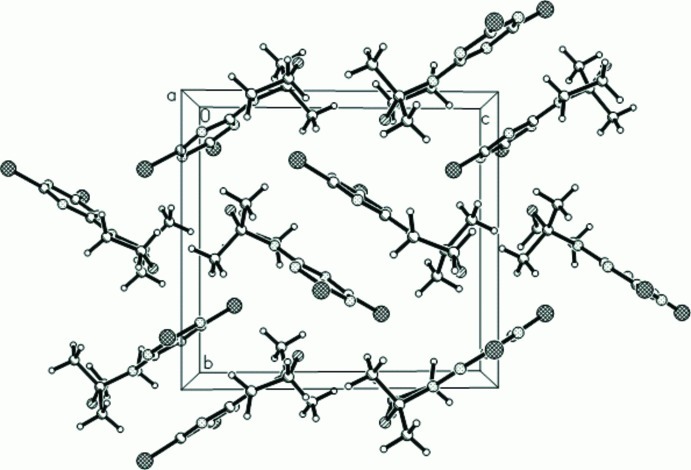
The crystal packing of the title compound, viewed down the a axis of the unit cell.

## supplementary crystallographic information

### Comment

2,4,6-Trichloro-1,3,5-triazine, because of the excellent and different reactivity of each chlorine atom, can react with organic amines or compounds containing active hydrogen to form compounds that have various substituent groups (Li *et al.*, 2010); Xue *et al.*, 2011; Zhao *et al.*, 2010). The Aldol reaction is also particularly useful in organic synthesis for the facile formation of C—C bonds. A similar mechanism to that of the Aldol reaction is involved in the reaction of acetone with *N*-yl-2-iminoethanol (Yang *et al.*, 2010; Rankin *et al.*, 2002). The title compound C_8_H_10_Cl_2_N_4_O was the product from a combination of such reactions and the structure is reported here.

In this compound (Fig. 1), the dichloro-substituted triazine ring and the quasi-plane of the five-membered dimethyl-substituted oxazolidine moiety, in which the O-atom lies 0.228 (1) Å out of the l.s. plane, are close to coplanar [dihedral angle, 4.99 (10)°]. An intramolecular methyl C—H···N_triazine_ interaction is present. The crystal packing is stabilized by a single intermolecular C2—H···Cl2^i^ interaction (Table 1), giving chains which extend along *a* (Fig. 2). Also present are weak π–π interactions between triazine rings [minimum ring centroid separation, 3.7427 (11) Å].

### Experimental

The title compound was prepared in a two-step synthesis: 1:1 Stoichiometric quantities of 2,4,6-trichloro-1,3,5-triazine and ethanolamine were first reacted in an ice bath (Xue *et al.*, 2011). The intermediate product from the first step was then reacted with acetone in the presence of base as a catalyst in an Aldol reaction (Yang *et al.*, 2010; Rankin *et al.*, 2002). Single crystals suitable for X-ray diffraction were obtained by evaporation of a solution of the title compound in toluene at room temperature.

### Refinement

All H atoms were positioned geometrically and treated as riding, with C—H bond lengths constrained to 0.98 Å (methyl) and 0.99 Å (methylene), and with *U*_iso_(H) = 1.2*U*_eq_ (methylene C) and 1.5*U*_eq_(methyl C).

### Crystal data

**Table:**

C_8_H_10_Cl_2_N_4_O	*F*(000) = 512
*M**_r_* = 249.10	*D*_x_ = 1.542 Mg m^−^^3^
Monoclinic, *P*2_1_/*n*	Mo *K*α radiation, λ = 0.71073 Å
Hall symbol: -P 2yn	Cell parameters from 4131 reflections
*a* = 8.1943 (10) Å	θ = 1.7–27.9°
*b* = 11.0948 (17) Å	µ = 0.58 mm^−^^1^
*c* = 11.8333 (18) Å	*T* = 113 K
β = 94.383 (14)°	Plate, colorless
*V* = 1072.7 (3) Å^3^	0.20 × 0.20 × 0.06 mm
*Z* = 4	

### Data collection

**Table:**

Rigaku Saturn724 CCD-detector diffractometer	2547 independent reflections
Radiation source: rotating anode	1592 reflections with *I* > 2σ(*I*)
Multilayer monochromator	*R*_int_ = 0.058
Detector resolution: 14.22 pixels mm^-1^	θ_max_ = 27.8°, θ_min_ = 2.5°
ω and φ scans	*h* = −10→10
Absorption correction: multi-scan (*SADABS*; Sheldrick, 1996)	*k* = −14→14
*T*_min_ = 0.892, *T*_max_ = 0.966	*l* = −15→15
13220 measured reflections	

### Refinement

**Table:**

Refinement on *F*^2^	Primary atom site location: structure-invariant direct methods
Least-squares matrix: full	Secondary atom site location: difference Fourier map
*R*[*F*^2^ > 2σ(*F*^2^)] = 0.038	Hydrogen site location: inferred from neighbouring sites
*wR*(*F*^2^) = 0.088	H-atom parameters constrained
*S* = 0.97	*w* = 1/[σ^2^(*F*_o_^2^) + (0.0381*P*)^2^] where *P* = (*F*_o_^2^ + 2*F*_c_^2^)/3
2547 reflections	(Δ/σ)_max_ = 0.001
138 parameters	Δρ_max_ = 0.40 e Å^−^^3^
0 restraints	Δρ_min_ = −0.40 e Å^−^^3^

### Special details

**Table:**

Geometry. All e.s.d.'s (except the e.s.d. in the dihedral angle between two l.s. planes) are estimated using the full covariance matrix. The cell e.s.d.'s are taken into account individually in the estimation of e.s.d.'s in distances, angles and torsion angles; correlations between e.s.d.'s in cell parameters are only used when they are defined by crystal symmetry. An approximate (isotropic) treatment of cell e.s.d.'s is used for estimating e.s.d.'s involving l.s. planes.
Refinement. Refinement of *F*^2^ against ALL reflections. The weighted *R*-factor *wR* and goodness of fit *S* are based on *F*^2^, conventional *R*-factors *R* are based on *F*, with *F* set to zero for negative *F*^2^. The threshold expression of *F*^2^ > σ(*F*^2^) is used only for calculating *R*-factors(gt) *etc*. and is not relevant to the choice of reflections for refinement. *R*-factors based on *F*^2^ are statistically about twice as large as those based on *F*, and *R*- factors based on ALL data will be even larger.

### Fractional atomic coordinates and isotropic or equivalent isotropic displacement parameters (Å^2^)

**Table:**

	*x*	*y*	*z*	*U*_iso_*/*U*_eq_	
Cl1	0.76979 (6)	0.25777 (5)	−0.13199 (4)	0.02482 (15)	
Cl2	0.23647 (6)	0.16452 (5)	0.06027 (4)	0.02427 (15)	
O1	0.85464 (15)	−0.10346 (12)	0.36264 (11)	0.0234 (3)	
N1	0.77455 (18)	0.01989 (14)	0.21633 (12)	0.0186 (4)	
N2	0.76821 (19)	0.12912 (14)	0.05072 (13)	0.0183 (4)	
N3	0.51066 (18)	0.20524 (14)	−0.02873 (12)	0.0181 (4)	
N4	0.52080 (18)	0.09188 (15)	0.14388 (13)	0.0192 (4)	
C1	0.9986 (2)	−0.04035 (19)	0.33452 (16)	0.0256 (5)	
H1A	1.0933	−0.0957	0.3342	0.031*	
H1B	1.0256	0.0256	0.3891	0.031*	
C2	0.9532 (2)	0.00951 (19)	0.21618 (16)	0.0244 (5)	
H2A	1.0048	0.0889	0.2053	0.029*	
H2B	0.9849	−0.0467	0.1567	0.029*	
C3	0.7163 (2)	−0.03406 (17)	0.32222 (15)	0.0196 (4)	
C4	0.5752 (2)	−0.12112 (18)	0.30178 (17)	0.0250 (5)	
H4A	0.6003	−0.1792	0.2432	0.037*	
H4B	0.4758	−0.0764	0.2767	0.037*	
H4C	0.5579	−0.1642	0.3722	0.037*	
C5	0.6822 (2)	0.06585 (18)	0.40536 (16)	0.0273 (5)	
H5A	0.6539	0.0302	0.4771	0.041*	
H5B	0.5907	0.1153	0.3737	0.041*	
H5C	0.7798	0.1163	0.4189	0.041*	
C6	0.6855 (2)	0.08098 (17)	0.13663 (15)	0.0181 (4)	
C7	0.6724 (2)	0.18873 (17)	−0.02348 (16)	0.0184 (4)	
C8	0.4475 (2)	0.15203 (17)	0.05908 (16)	0.0182 (4)	

### Atomic displacement parameters (Å^2^)

**Table:**

	*U*^11^	*U*^22^	*U*^33^	*U*^12^	*U*^13^	*U*^23^
Cl1	0.0252 (3)	0.0285 (3)	0.0215 (3)	−0.0004 (2)	0.0066 (2)	0.0066 (2)
Cl2	0.0191 (3)	0.0262 (3)	0.0279 (3)	0.0008 (2)	0.0043 (2)	0.0039 (2)
O1	0.0207 (8)	0.0221 (8)	0.0271 (8)	0.0000 (6)	0.0000 (6)	0.0085 (6)
N1	0.0181 (9)	0.0191 (9)	0.0188 (9)	0.0009 (7)	0.0032 (7)	0.0050 (7)
N2	0.0205 (9)	0.0181 (9)	0.0167 (9)	−0.0003 (7)	0.0034 (7)	0.0010 (7)
N3	0.0209 (9)	0.0167 (9)	0.0168 (9)	0.0000 (7)	0.0017 (7)	−0.0003 (7)
N4	0.0184 (9)	0.0188 (9)	0.0206 (9)	0.0011 (7)	0.0024 (7)	0.0023 (7)
C1	0.0209 (11)	0.0289 (12)	0.0268 (12)	0.0012 (9)	0.0008 (9)	0.0063 (10)
C2	0.0207 (12)	0.0276 (12)	0.0254 (12)	0.0030 (9)	0.0046 (9)	0.0053 (9)
C3	0.0207 (11)	0.0206 (11)	0.0174 (10)	0.0003 (8)	0.0012 (8)	0.0040 (9)
C4	0.0267 (12)	0.0257 (12)	0.0222 (11)	−0.0075 (9)	0.0000 (9)	0.0054 (9)
C5	0.0307 (12)	0.0289 (12)	0.0229 (11)	0.0010 (10)	0.0047 (9)	−0.0022 (10)
C6	0.0229 (11)	0.0140 (10)	0.0177 (10)	−0.0018 (8)	0.0029 (8)	−0.0011 (8)
C7	0.0244 (12)	0.0156 (10)	0.0156 (10)	−0.0022 (8)	0.0037 (8)	−0.0031 (8)
C8	0.0195 (11)	0.0158 (10)	0.0194 (11)	−0.0016 (8)	0.0027 (8)	−0.0053 (8)

### Geometric parameters (Å, º)

**Table:**

Cl1—C7	1.7402 (19)	C1—C2	1.525 (2)
Cl2—C8	1.7360 (19)	C1—H1A	0.9900
O1—C3	1.422 (2)	C1—H1B	0.9900
O1—C1	1.432 (2)	C2—H2A	0.9900
N1—C6	1.333 (2)	C2—H2B	0.9900
N1—C2	1.469 (2)	C3—C4	1.512 (2)
N1—C3	1.499 (2)	C3—C5	1.522 (2)
N2—C7	1.311 (2)	C4—H4A	0.9800
N2—C6	1.372 (2)	C4—H4B	0.9800
N3—C8	1.333 (2)	C4—H4C	0.9800
N3—C7	1.335 (2)	C5—H5A	0.9800
N4—C8	1.312 (2)	C5—H5B	0.9800
N4—C6	1.364 (2)	C5—H5C	0.9800
			
C3—O1—C1	107.84 (14)	N1—C3—C5	109.60 (15)
C6—N1—C2	122.07 (16)	C4—C3—C5	113.10 (16)
C6—N1—C3	127.05 (16)	C3—C4—H4A	109.5
C2—N1—C3	110.53 (14)	C3—C4—H4B	109.5
C7—N2—C6	112.84 (16)	H4A—C4—H4B	109.5
C8—N3—C7	110.25 (16)	C3—C4—H4C	109.5
C8—N4—C6	113.21 (16)	H4A—C4—H4C	109.5
O1—C1—C2	104.09 (15)	H4B—C4—H4C	109.5
O1—C1—H1A	110.9	C3—C5—H5A	109.5
C2—C1—H1A	110.9	C3—C5—H5B	109.5
O1—C1—H1B	110.9	H5A—C5—H5B	109.5
C2—C1—H1B	110.9	C3—C5—H5C	109.5
H1A—C1—H1B	109.0	H5A—C5—H5C	109.5
N1—C2—C1	101.55 (15)	H5B—C5—H5C	109.5
N1—C2—H2A	111.5	N1—C6—N4	119.43 (17)
C1—C2—H2A	111.5	N1—C6—N2	116.57 (17)
N1—C2—H2B	111.5	N4—C6—N2	124.00 (17)
C1—C2—H2B	111.5	N2—C7—N3	129.93 (18)
H2A—C2—H2B	109.3	N2—C7—Cl1	115.56 (14)
O1—C3—N1	101.59 (14)	N3—C7—Cl1	114.51 (14)
O1—C3—C4	106.75 (16)	N4—C8—N3	129.73 (18)
N1—C3—C4	114.19 (15)	N4—C8—Cl2	115.62 (14)
O1—C3—C5	110.98 (15)	N3—C8—Cl2	114.65 (14)
			
C3—O1—C1—C2	−39.6 (2)	C2—N1—C6—N2	3.1 (3)
C6—N1—C2—C1	167.26 (17)	C3—N1—C6—N2	175.65 (16)
C3—N1—C2—C1	−6.4 (2)	C8—N4—C6—N1	−178.75 (17)
O1—C1—C2—N1	26.7 (2)	C8—N4—C6—N2	0.9 (3)
C1—O1—C3—N1	34.21 (18)	C7—N2—C6—N1	−179.35 (17)
C1—O1—C3—C4	154.11 (15)	C7—N2—C6—N4	1.0 (3)
C1—O1—C3—C5	−82.24 (18)	C6—N2—C7—N3	−2.3 (3)
C6—N1—C3—O1	170.59 (17)	C6—N2—C7—Cl1	177.47 (13)
C2—N1—C3—O1	−16.15 (19)	C8—N3—C7—N2	1.4 (3)
C6—N1—C3—C4	56.1 (3)	C8—N3—C7—Cl1	−178.37 (13)
C2—N1—C3—C4	−130.64 (18)	C6—N4—C8—N3	−2.1 (3)
C6—N1—C3—C5	−72.0 (2)	C6—N4—C8—Cl2	177.73 (14)
C2—N1—C3—C5	101.30 (18)	C7—N3—C8—N4	1.1 (3)
C2—N1—C6—N4	−177.19 (17)	C7—N3—C8—Cl2	−178.75 (13)
C3—N1—C6—N4	−4.7 (3)		

### Hydrogen-bond geometry (Å, º)

**Table:**

*D*—H···*A*	*D*—H	H···*A*	*D*···*A*	*D*—H···*A*
C2—H2*A*···Cl2^i^	0.99	2.78	3.522 (2)	132
C4—H4*B*···N4	0.98	2.49	3.025 (3)	114

Symmetry code: (i) *x*+1, *y*, *z*.
